# Innate Lymphoid Cells in Autoimmune Diseases

**DOI:** 10.3389/fimmu.2021.789788

**Published:** 2022-01-07

**Authors:** Aurelie S. Clottu, Morgane Humbel, Natalia Fluder, Maria P. Karampetsou, Denis Comte

**Affiliations:** ^1^ Service of Immunology and Allergy, Lausanne University Hospital, University of Lausanne, Lausanne, Switzerland; ^2^ Division of Rheumatology, Evaggelismos General Hospital, Athens, Greece

**Keywords:** innate lymphoid cell (ILC), autoimmune diseases, autoimmunity, systemic lupus erythematosus, systemic sclerosis, rheumatoid arthritis, ANCA-associated vasculitis, NK cell

## Abstract

Innate lymphoid cells (ILC) are a heterogeneous group of immune cells characterized by lymphoid morphology and cytokine profile similar to T cells but which do not express clonally distributed diverse antigen receptors. These particular cells express transcription factors and cytokines reflecting their similarities to T helper (Th)1, Th2, and Th17 cells and are therefore referred to as ILC1, ILC2, and ILC3. Other members of the ILC subsets include lymphoid tissue inducer (LTi) and regulatory ILC (ILCreg). Natural killer (NK) cells share a common progenitor with ILC and also exhibit a lymphoid phenotype without antigen specificity. ILC are found in low numbers in peripheral blood but are much more abundant at barrier sites such as the skin, liver, airways, lymph nodes, and the gastrointestinal tract. They play an important role in innate immunity due to their capacity to respond rapidly to pathogens through the production of cytokines. Recent evidence has shown that ILC also play a key role in autoimmunity, as alterations in their number or function have been identified in systemic lupus erythematosus, systemic sclerosis, and rheumatoid arthritis. Here, we review recent advances in the understanding of the role of ILC in the pathogenesis of autoimmune diseases, with particular emphasis on their role as a potential diagnostic biomarker and as therapeutic targets.

## Introduction

Innate lymphoid cells (ILC) are lymphocytes that lack somatically diversified antigen receptor expression (1). So far, different subtypes of ILC have been described, depending on their specific functional features mirroring CD4^+^ T helper (Th)1, Th2, and Th17 cells. In 2013, they were classified as group 1 (including NK cells), group 2, and group 3 ILC (1, 2); however, following further research, a new description was proposed in 2018 which classifies ILC into five categories, namely, NK cells, ILC1, ILC2, ILC3, and lymphoid tissue inducer (LTi) cells (**Table 1**) (3).

**Table 1:** Characteristics of innate lymphoid cell (ILC) populations in humans.

		ILC1	ILC2	ILC3	LTi	ILCreg	NK
Function		Antimicrobial defense (intracellular microbes) (1)	Allergies, parasite elimination (3)	Innate immunity against fungi and extracellular microbes (1, 3)	Mesenchymal organizer for SLN in embryonic development (3)	Resolution of innate intestinal inflammation (4)	Antitumor surveillance, antimicrobial defense (intracellular pathogens and viruses) (5)
Phenotype		Variable depending on tissue residency	CRTH2^+^ST2^+^CD161^+^ (6, 7)	Controversial Lin^−^ (CD56^+/−^)CD127^+^CRTH2^−^CD117^+^NKp44^+/−^ (8, 9)	Controversial NRP1 (10)	Lin^−^CD45^+^CD127^+^IL10^+^ (11)	CD56^+^CD16^+/−^
Transcription factor		T-bet (1, 3, 12)	GATA3, RORα (1, 3, 13)	RORγt (1, 3)	RORγt (3)	–	T-bet, EOMES (3, 5, 12, 14, 15)
Inducing cytokines		IL-12 (16)	IL-33, IL-25, TSLP (1, 7, 17)	IL-7, IL-23, IL-1β (3, 18)	–	–	IL-15 (3, 5, 12, 14, 15)
Produced cytokines		IFN-γ (1, 3, 12)	IL-4, IL-5, IL-13 (1, 7, 17)	IL-22, IL-17A, GM-CSF, IFN-γ, TNF-α (3, 18)	IL-22, IL-17, GM-CSF, TNF-α and TNF-β, IL-8 (10)	IL-10, TGF-β (11)	IFN-γ, perforin granzyme B
Regulated tissues	Main	Tonsils, gut, lung, liver, adipose tissue, skin, LN, spleen (6, 19–21)	Peripheral blood, skin, lungs, adipose tissue (6, 7, 22)	Mucosal tissues (gut) (6, 16)	Lymphoid organs	ILCreg: intestine (11), kidney (23); follicular ILCreg: tonsils, LN (24)	Blood
	Possible	Peripheral/cord blood (6)	Liver, LN, spleen, adenoids (6)	Blood, spleen, LN, tonsils, intestine, skin and lung (6)	–	–	
Association with systemic autoimmune diseases		Increased in Ssc, SLE, active AAV (25–31), Increased or decreased in RA (32, 33)	Increased in SSc and RA (33–35), Decreased in AAV and SLE (27–29, 31)	Increased (29) or decreased (27, 28) in SLE, Increased (36) or decreased (33) in RA Decreased in AAV (31)	Decreased in RA (32)		

IL, interleukin; NRP1, neuropilin1; T-bet, T box expressed in T cells; GATA 3, Trans-acting T-cell-specific transcription factor GATA3; RORγT, retinoid acid-related orphan receptor γ T; RORα, retinoid acid-related orphan receptor α; LN, lymph node; CRTH2, chemoattractant-homologous receptor expressed on Th2 cells; Ssc, systemic sclerosis; SLE, systemic lupus erythematosus; AAV, ANCA-associated vasculitis; RA, rheumatoid arthritis.

ILC in humans and mice originate from a common lymphoid precursor (CLP), which is able to give birth to all lymphocyte subsets (37). Studies in murine models have shown that CLP initially differentiates into the common innate lymphoid progenitor (CILP) which serves as a common precursor for both NK cells and ILC. CILP then evolves into the common helper innate lymphoid progenitor (CHILP), which is common to LTi and ILC (6, 38). CHILP finally differentiates into innate lymphoid cell precursors (ILCP) that will give rise to ILC1, ILC2, and ILC3 (3, 12). Of note, in mice, lymphoid progenitors (which have the potential to differentiate into all ILC lineages, including NK cells) were identified as inhibitor of DNA binding 2 (ID2) positive (5). ID2 is a transcription factor required for organogenesis of lymphoid tissues, and its loss was shown to disrupt the generation of ILC precursors (14, 15). In humans, the differentiation steps that lead to the development of ILC are less well known even though they are considered to be similar (19). Similarities and differences between human and murine ILC have been excellently reviewed elsewhere (19).

Many phenotypic markers have been used to characterize mature ILC subsets, but no definitive marker universally defining ILC has been identified so far. This is notably due to the fact that their phenotype depends on the tissue they populate and that ILC represent very heterogeneous populations (20, 21). Despite tremendous variability in their definition, ILC can be roughly described as CD3-negative lymphocytes that express IL-7 receptor (CD45^+^CD3^−^CD127^+^), although in many tissues ILC1 do not express CD127 (20, 21).

NK cells were initially included in group 1 ILC, together with ILC1, because of important similarities such as the expression of the transcription factor T-bet and the production of interferon γ (IFN-γ) (3, 7). However, subsequent studies indicated that NK cells and ILC1 belong to distinct lineages and represent two separate cell types (6, 13, 17, 21). Indeed, while ILC are mainly tissue-resident cells, NK cells are principally found in blood circulation, constituting 5%–20% of circulating lymphocytes, and are capable of being rapidly recruited to inflammation sites (20, 39, 40). Moreover, NK cells have an important cytotoxic function with high expression levels of perforin and granzyme B, whereas ILC1 are in general noncytotoxic or only weakly cytotoxic. Interestingly, recent evidence in murine models shows that CD160^-^ILC1 exhibit cytotoxicity against YAC-1 cells (22). In addition, certain populations of splenic ILC1-like NK cells are able to kill cells infected with murine cytomegalovirus (3, 18, 22, 39). NK cells play a special role in antitumor surveillance and in antimicrobial defense against intracellular pathogens and viruses (39). In addition, compared with ILC1, they follow a specific differentiation pathway that requires the expression of the T-box transcription factor Eomes for their development, and the induction of CD122 with subsequent IL-15 responsiveness (8, 13, 16).

In addition, ILC1 also share similarities with Th1 cells, as they react to intracellular pathogens, mainly secrete IFN-γ and depend on the transcription factor T-bet for their differentiation (1, 3, 6). Although they can be detected in peripheral blood or cord blood, they are primarily tissue-resident cells (20). In humans, ILC1 are mainly found in the tonsils, gut, lung, liver, adipose tissue, skin, lymph nodes, and spleen (4, 9, 20, 40). They show significant differences in the expression of surface markers and transcription factors linked to the microenvironment of the tissue they populate (20).

ILC2, like Th2 cells, produce high levels of interleukin (IL)-4, IL-5, and IL-13 in response to epithelial cell-derived IL-33, IL-25, and thymic stromal lymphopoietin (TSLP) (1, 10, 41). They express high levels of the transcription factors GATA3 and RORα (3, 11). Phenotypically, they are characterized, in mice, by the expression of suppression of tumorigenicity (ST2, also known as IL-1RL1) (23, 24), CD161, and inducible T-cell COStimulator (ICOS), whereas in human peripheral blood, they are described as chemoattractant-homologous receptor expressed on Th2 cells (CRTH2^+^), ST2^+^, and/or CD161^+^ (20, 41). ILC2 are involved in allergies and parasite elimination (3).

In mice, they are abundant in the airways, lungs, skin, and gut, especially in models of asthma (1, 41, 42). In humans, ILC2 represent the main population of ILC that inhabit peripheral blood, skin, lungs, and adipose tissue, but they are little or not present in adult gut (20, 41, 43). Furthermore, the presence of ILC2 has also been described in liver, lymph nodes, spleen, and adenoids (20).

ILC3 are the innate counterpart of Th17 cells. They play a role in innate immunity against fungi and extracellular microbes and depend on the transcription factor RORγt (1, 3). Like other ILCs, they require IL-7 for their development. More specifically, they secrete IL-22 and certain subsets can produce IL-17A, in response to IL-23 and IL-1β (3, 44). In mouse models, ILC3 participate in the secondary antibody response by promoting the survival of CD4^+^ T cells through the expression of OX40 ligand and CD30 ligand (44). They are also able to express antigen-presenting molecule major histocompatibility complex-II (MHC-II) and present processed antigens to CD4^+^ T cells (44).

In humans, ILC3 and notably NKp44^+^ ILC3 are particularly found in mucosal tissues such as the gut (20, 45). However, they may also be found in blood, spleen, lymph nodes, tonsils, skin, and lung (20). Phenotypically, in humans, they were notably described as Lin^−^CD56^+/−^CD127^+^CRTH2^−^CD117^+^NKp44^+/−^ (46) or Lin^−^CD45^+^CD127^+^cKit^+^CRTH2^−^NKp44^−^ or NKp44^+/−^ (47).

LTi were previously included in the group 3 ILC because of their capacity to produce IL-17 and IL-22. They undergo differentiation from a specific progenitor, the lymphoid tissue inducer progenitor (LTiP) and depend on RORγt for their differentiation (3). However, now considered a specific population, LTi have a specific role as mesenchymal organizer cells in the formation of secondary lymphoid structures during embryonic development (3). According to data from studies in mice, the crosstalk between LTi and lymphoid tissue stromal cells continues postnatally, as it has been demonstrated that LTi cells contribute to the restoration of lymphoid tissue architecture following infection with LCMV (48). In humans, LTi express neuropilin-1, produce IL-17, IL-22, GM-CSF, TNF-α, TNF-β, and IL-8, and play possibly a role in the Th1 and Th17 immune response (49, 50).

Lately, another ILC subpopulation was described, which harbors a regulatory phenotype, and hence named regulatory ILC (ILCreg) (51). These cells, phenotypically defined as Lin^−^CD45^+^CD127^+^IL-10^+^, were initially described in mouse and human intestine secrete high amounts of IL-10 and TGF-β and are devoid of CD4 and Foxp3 expression (51). They show a distinct gene expression profile compared with other ILC and play an important role in the resolution of innate intestinal inflammation through the suppression of ILC1 and ILC3 *via* IL-10 secretion, in a mouse model of colitis (51). In addition, the secretion of TGF-β acts in an autocrine way to support the expansion of ILCreg during gut inflammation (51). Of note, the existence of IL-10-producing ILCreg as a distinct population of ILC remains controversial. From this point of view, in various mice models, the main source of IL-10 in the gastrointestinal tract comes from activated populations of ILC2, which expresses KLRG1, IL-25R, and the transcription factor GATA-3 (52).

In another context, ILCreg were also described in mouse and human kidney, where they play a protective role in ischemia-reperfusion injury (53). Another regulatory population of ILC, named follicular regulatory ILC, has been described in human tonsils and lymph nodes and secretes high amounts of TGF-β (54).

Interestingly, ILC have been recently shown to exhibit plasticity, similarly to T cells. They have the ability to coexpress lineage-determining transcription factors in response to signal from their microenvironment (23). This is especially true for CD127^+^CD117^+^ ILC precursors, a cell subset which expresses CD45RA and CD62L and shows similarities to naive CD4^+^ T cells (23). Their differentiation depends on cytokines present in the tissue they populate (23). Balance between ILC1 and ILC3 changes in the presence of inflammatory stimulations, with ILC1 numbers increasing and ILC3 decreasing in the intestine in pathological conditions such as Crohn’s disease (45). This process occurs through a differentiation of ILC3 to ILC1, which depends on exposure to IL-12. In addition, this differentiation has been shown to be reversible, as the presence of IL-23 and IL-1β favors the differentiation of ILC1 to ILC3 (45). Recently, ILC3–ILC1 intermediate subsets were identified in human tonsils and intestinal mucosa, describing ILC3 and ILC1 as the ends of a spectrum, with the cells closest to ILC1 having the maximal ability to produce IFN-γ *in vitro* (4). Another study showed that human ILC3 that are transferred to humanized mice acquire ILC1-like features in the spleen more than in the liver (5). These results support the hypothesis that tissue specific triggers cause local transdifferentiation of ILC (23).

Since their discovery, numerous studies suggest that ILC play a key role in the pathogenesis of systemic autoimmune conditions such as systemic sclerosis, systemic lupus erythematosus, rheumatoid arthritis, and antineutrophil cytoplasm antibody (ANCA)-associated vasculitides. In this review, we discuss the latest advances on the role of ILC in the pathogenesis of human autoimmune diseases and their potential use as diagnostic biomarkers and/or therapeutic targets.

## ILC in Autoimmune Conditions

### Systemic Sclerosis

Systemic sclerosis (Ssc) is an autoimmune connective tissue disease characterized by vasculopathy and fibrosis in multiple organs. Ssc prototypically causes Raynaud phenomenon, arthralgias, fingertip lesions, skin thickening, hypertensive renal crisis, lung fibrosis, and pulmonary arterial hypertension (55–57). The pathophysiology of Ssc is unclear but involves genetic and environmental factors (i.e., silica solvents, epoxy resins, breast implants, skin microbiota), leading to chronic inflammation, endothelial injury, vascular dysfunction, fibroblast activation, and tissue fibrosis (58, 59). Numerous immune cells, antibodies, and cellular pathways contribute to the processes that lead to tissue fibrosis. In particular, dysregulation of interferon α (IFN-α) is an important alteration in patients with antitopoisomerase I antibodies (58, 60). This dysregulation is characterized by an IFN-α overproduction by plasmacytoid dendritic cells in response to the activation of toll-like receptors (TLR) 7 and 9 by immune complexes generated by endothelial cell death (60). Constitutive fibroblast activation driven by mediators such as tumor growth factor β (TGF-β) also represents a key process, which leads to tissue fibrosis (58).

**Figure 1:**
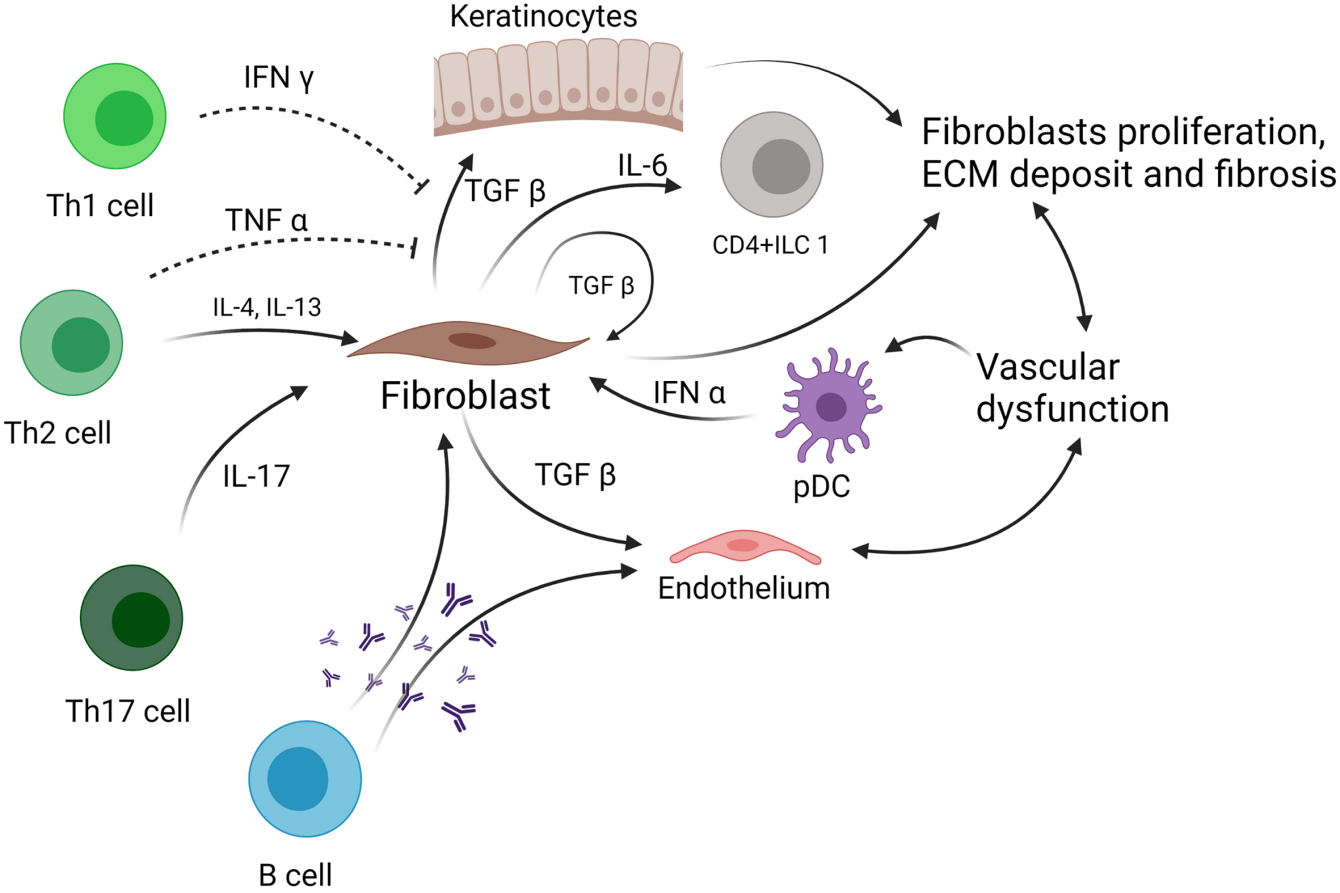
Role of ILC in systemic sclerosis. ILC, innate lymphoid cell; TNF-α, tumor necrosis factor α; TGF-β, tumor growth factor β; pDC, plasmacytoid dendritic cell; IL, interleukin; ECM, extracellular matrix.

Few studies have examined the role of ILCs in the pathophysiology of human Ssc (**Figure 1**). A study published in 2015 by Wohlfahrt et al., including 69 Ssc patients, showed that ILC2 number is elevated in the skin and peripheral blood of patients with Ssc compared with healthy controls (34). There was also a positive correlation between the number of ILC2 in the skin and the modified Rodnan Skin Score. In addition, patients with extensive pulmonary fibrosis showed the highest numbers of circulating ILC2 (34). Of note, ILC2 were defined using two different marker panels, both including ST2 (ICOS^+^ST2^+^CD3^−^CD11b^−^ or ST2^+^IL-17RB^+^KLRG1^+^), with consistent results (34). These data suggest a potential pathogenic role of ILC2 in Ssc, although the mechanism is still unclear. As it was shown that type 2 cytokines such as IL-4 and IL-13 can increase TGF-β production in bronchial epithelial cells in diseases such as asthma (61), one could hypothesize that ILC2, which secrete such cytokines, could thus induce TGF-β secretion from fibroblasts or other epithelial cells such as keratinocytes, and therefore, increase fibrosis (62). Moreover, in murine models, TGF-β is required for the development of ILC2, suggesting a potential crosstalk between fibroblasts and ILC2 (63). However, data are still missing in Ssc, and this hypothesis needs to be investigated.

On the other hand, a study published in 2016 by Roan et al. showed that a subset of ILC1, defined as CD4^+^ ILC1, and NKp44^+^ ILC3 were increased in the peripheral blood of Ssc patients compared with healthy subjects (25, 26). An interesting point is that the CD4^+^ ILC1 expressing IL-6Rα were decreased in SSc, suggesting that these cells are overactivated and contribute to the amplification of the inflammatory response that characterizes SSc (25, 26). In another study, the authors showed that KLRG1^low^ ILC2 are increased in the fibrotic skin from SSc patients. This population is activated by TGF-β and produces lower levels of IL-10 compared with KLRG1^high^ ILC2. These KLRG1^low^ ILC2 cells fail to negatively regulate collagen production by dermal fibroblast, a process which is physiologically IL-10 dependent, thus enhancing skin fibrosis (35). Despite these interesting findings on the role of ILC1 and ILC2 in Ssc pathogenesis and fibrosis development, data are still missing to fully understand the importance of ILC in the pathogenesis of Ssc.

### Systemic Lupus Erythematosus

Systemic lupus erythematosus (SLE) is a multisystem autoimmune disease affecting mainly young women of childbearing age. Its pathophysiology is complex, involving loss of self-tolerance with an imbalance between apoptotic cell abundance, extracellular exposition of nuclear antigens, and disposal of this apoptotic material. The free nuclear antigens will activate TLR notably on plasmacytoid dendritic cells (pDC), with secretion of type I IFN (known as the “interferon signature”) and other cytokines that drive B-cell differentiation, and the production of autoantibodies (64). These antibodies directed against self-antigens then form immune complexes that deposit in the tissues, leading to chronic inflammation and tissue damage (27, 64).

**Figure 2:**
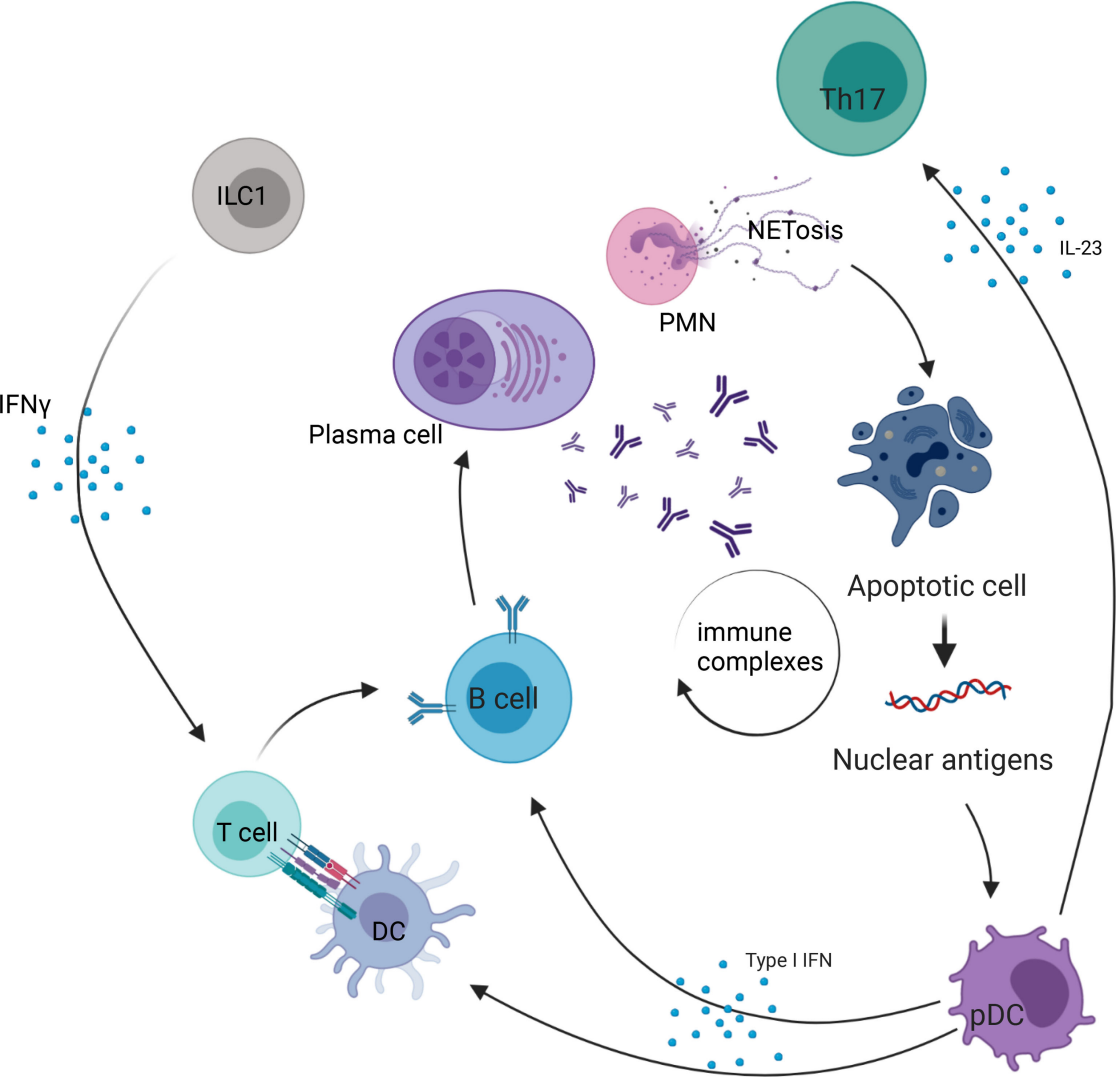
Role of ILC in systemic lupus erythematosus. PMN, polymorphonuclear cell; ILC, innate lymphoid cell; NETosis, process of neutrophil extracellular traps; IL, interleukin; DC, dendritic cell; pDC, plasmacytoid dendritic cell; Th17, T helper 17 cell; IFN-γ, interferon γ.

The role of ILC in SLE pathogenesis is poorly understood, particularly in humans (**Figure 2**). In 2019, a study by Guo et al. examined circulating ILC in the peripheral blood of 49 SLE patients and showed an increase in ILC1 (defined as Lin^−^CD127^+^CRTH2^−^CD117^−^) compared with healthy controls, while ILC2 (Lin^−^CD127^+^CRTH2^+^) and ILC3 (including 2 subpopulations, defined as Lin^−^CD127^+^CRTH2^−^CD117^+^NKp44^+^ or NKp44^−^) were decreased (28). Moreover, the greatest increase in ILC1 and decrease in ILC2 and ILC3 were observed in patients with moderate and severe disease activity, with a positive correlation of ILC1 numbers to systemic lupus erythematosus disease activity index (SLEDAI) (28). This altered distribution of ILC in active SLE with lupus nephritis was reversed after initiation of treatment (steroids and cyclophosphamide), suggesting that ILC1 may represent a biomarker of disease activity (28). Recently, a study by Jiang et al. examined the number of ILC in the peripheral blood of SLE patients (29). They also found an increase in ILC1 and a decrease in ILC2 in patients with active SLE, but, in contrast to Guo et al., they found an increase of ILC3 in the blood of patients with active SLE compared with inactive (SLEDAI<5). Interestingly, there was a positive correlation between ILC3 absolute numbers in the peripheral blood and the SLEDAI score. This discrepancy between the two studies might be due to differences in gating used to define ILC subsets, as the markers used to distinguish between ILC1 and ILC3 were similar. Heterogeneity of SLE patients might also contribute to such differences. An interesting point in the research by Jiang et al. is a positive correlation between ILC3 and serum anti-dsDNA titers, and a decrease in ILC1/ILC3 and ILC2/ILC3 ratio in SLE patients with arthritis compared with patients without arthritis (29).

In a study by Blokland et al., which also included patients with primary Sjögren’s syndrome (pSS) patients, ILC1 were increased in the peripheral blood of SLE patients (27). In pSS, the abundance of total ILC did not differ from healthy donors but was associated with disease activity as measured by the EULAR Sjögren’s syndrome disease activity index (ESSDAI). The patients (SLE and pSS) showing an interferon (IFN) signature (defined by an elevated IFN score) had an increased FAS expression, with a decrease in ILC2 and ILC3 frequency (27).

Finally, a study including 51 SLE patients also showed an increase in ILC1 in the peripheral blood. They also identified a positive correlation between increased ILC1/ILC3 count and disease activity (30). These data suggest that ILC1 may participate in/constitute a response to the inflammatory process, while ILC3 may play a role in the development of the autoantibody response in SLE. However, further studies are warranted to explore these hypotheses and understand the role of the altered abundance of ILC in the peripheral blood of SLE patients. Moreover, in humans, ILC1 definition is still controversial (65), and no data are currently available on ILC phenotype in the organs and tissue from SLE patients. This would be of paramount importance to shed light on the role of these cells at the epithelial barrier sites in SLE.

### Antineutrophil Cytoplasm Antibody-Associated Vasculitis

ANCA-associated vasculitis (AAV) encompasses three distinct entities: granulomatosis with polyangiitis (GPA), microscopic polyangiitis (MPA), and eosinophilic granulomatosis with polyangiitis (EGPA) (66).

These inflammatory diseases are all characterized by small- and medium-vessel inflammation, but with relatively distinct clinical presentations, specific biologic features, and ANCA serotype. Anti-PR3 are mainly associated with GPA, while anti-MPO are more frequently associated with EGPA and MPA (67). GPA often manifests as granulomatous inflammation of the upper and lower airways and ear/nose granulomatous inflammation and kidney damage. MPA is characterized by necrotizing glomerulonephritis and pulmonary capillaritis. EGPA is prototypically associated to eosinophilia, pulmonary infiltrates, and asthma (68).

The pathogenesis of AAV relies on the production of antibodies that target myeloperoxydase and proteinase 3 (67). These two proteins are abnormally overexpressed on the surface of neutrophils, and, subsequently to antibody binding, neutrophils are activated and produce cytokines, reactive oxygen species, and neutrophil extracellular traps (NETosis) (67). Overactivation of B and T cells is also involved in the pathogenesis of AAV and leads to the production of ANCA (67).

**Figure 3:**
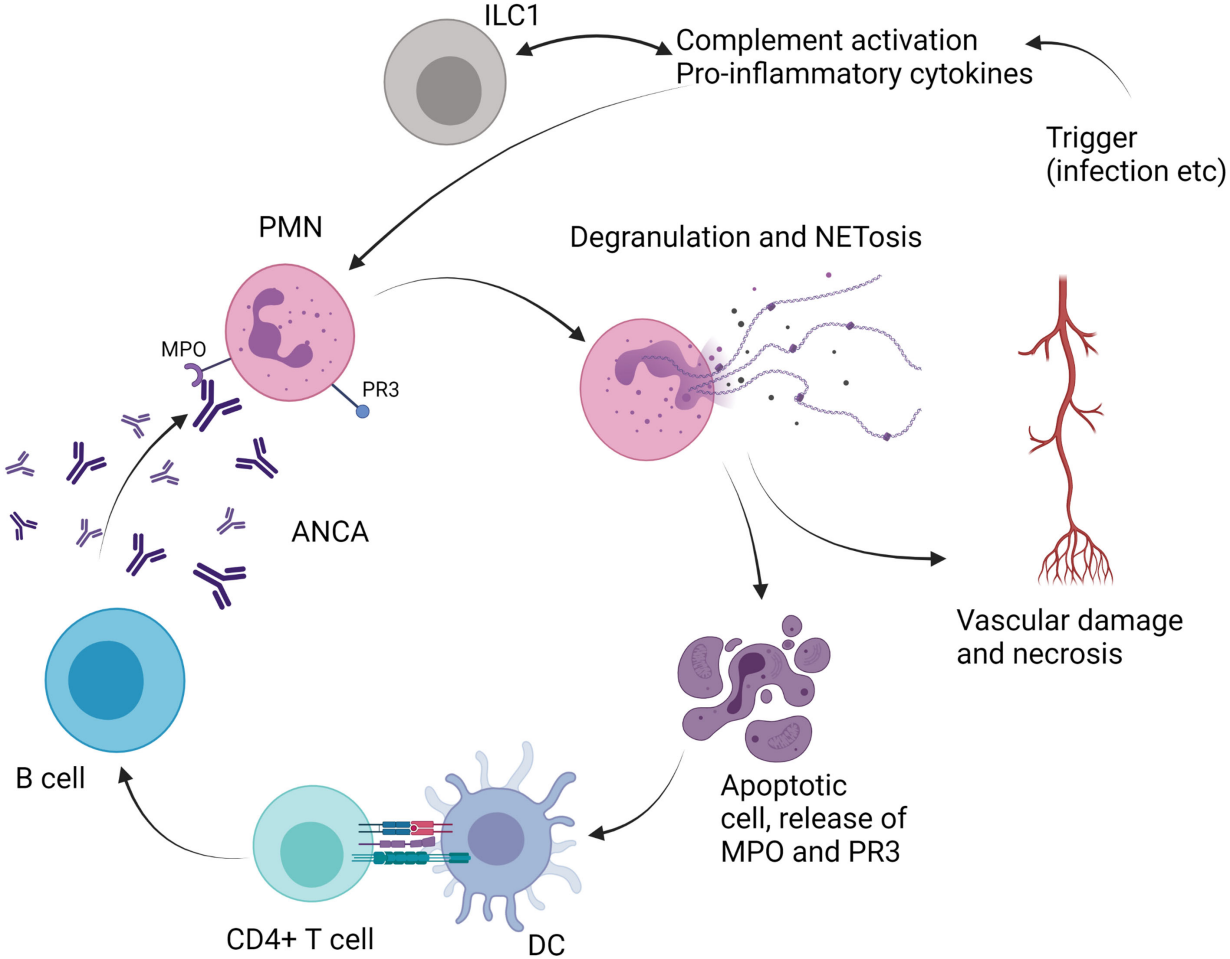
Role of ILC in antineutrophil cytoplasm antibodies (ANCA)-associated vasculitis. PMN, polymorphonuclear cell; ILC, innate lymphoid cell; DC, dendritic cell; MPO, myeloperoxidase; PR3, proteinase 3; NETosis, neutrophil extracellular traps; IL, interleukin.

As ILC have been shown to play particularly a role in tissue homeostasis at mucosal sites, and especially at the level of airways epithelia, examination of their role in AAV is of particular interest (**Figure 3**). From this point of view, one study examined the frequencies of ILCs in the peripheral blood of AAV patients (26 GPA and 15 MPA subjects) compared with healthy controls (31). Samples were collected during acute phase, defined by Birmingham vasculitis activity (BVAS) score >3, before any treatment, or during remission phase, defined as BVAS 0. Total ILCs, defined as Lin^−^CD127^+^, were decreased during acute phase in AAV patients compared with controls. More precisely, ILC2 and ILC3 were decreased while ILC1 were increased when compared with healthy controls or AAV patients in remission (31). Even if these data are of interest, it remains difficult to draw any definitive conclusion on the role of ILC in the pathogenesis of AAV. Further studies are warranted to address this point.

### Rheumatoid Arthritis

Rheumatoid arthritis (RA) is an autoimmune disease affecting the joints with synovial inflammation and cartilage/bone destruction (69). The pathogenesis is complex and involves the development of auto-antibodies such as rheumatoid factor (RF) and anti-citrullinated protein antibodies (ACPA), which can be detected years before the onset of clinical disease (70). Development of ACPA and/or RF is triggered by a complex interplay between genetic, epigenetic, and environmental factors (smoking, pathogens, obesity, dysbiosis, toxic substances) (69, 71, 72). Innate immunity is central to the pathogenesis of RA, with the presence of macrophages, mast cells, and NK cells in the synovial membrane, and neutrophils in the synovial fluid (69). NK cells are also increased in the synovial fluid of RA patients (73).

The humoral immune response plays also an essential role in the pathogenesis of RA, and B cells, plasmablasts, and plasma cells are very abundant in the inflamed synovium (69). ACPA promote the production of TNF-α by macrophages, a cytokine that is the cornerstone of RA joint damage, by activating fibroblasts and chondrocytes (69). Additionally, cytokines involved in the Th17 response, including IL-6, IL-21, IL-17, IL-23, and IL-1β, are also elevated in the peripheral blood and synovial fluid of patients with RA (71, 74).

**Figure 4:**
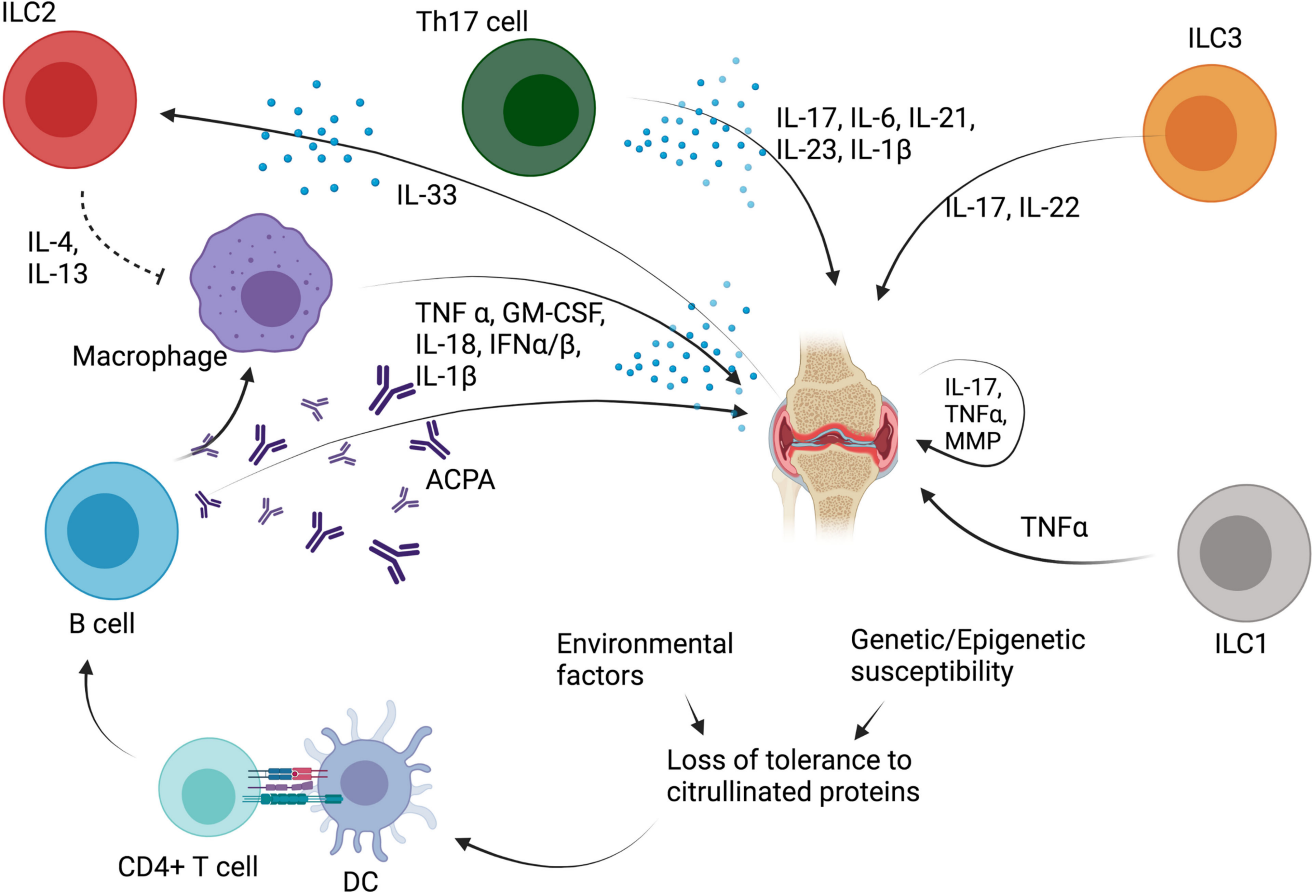
Role of ILC in rheumatoid arthritis (RA). ACPA, anticitrullinated protein antibodies; TNF-α, tumor necrosis factor α; IL, interleukin; DC, dendritic cell; MMP, metalloproteinase; ILC, innate lymphoid cell; IL, interleukin; GM-CSF, granulocyte-macrophage colony stimulating factor; IFN, interferon; Th, T helper CD4^+^ T cell.

Some recent studies evaluated the role of ILC in RA patients (**Figure 4**). In 2017, Rodriguez-Carrio et al. showed that ILC distribution differed in lymph nodes (LN) of RA patients compared with at-risk patients (defined as patients with RF and/or ACPA positivity, and arthralgia without arthritis) or healthy controls (32). LTi cells were shown to be decreased in RA patients, while ILC1 were increased in RA and at-risk patients. ILC3 were increased in RA patients compared with healthy controls and at-risk patients. A positive association of LTi frequency with VCAM expression on LN endothelial cells was also shown, suggesting a potential crosstalk between ILC and the stromal cell compartment (32). In 2019, Takaki-Kuwara et al. found that a subset of CCR6^+^ ILC3 was increased in the synovial fluid of RA patients compared with osteoarthritis controls, and positively correlated with RA clinical activity (36). Moreover, a positive correlation was established between the number of CCR6^+^ ILC3 cells and CCL20 concentration in synovial fluid of RA patients, suggesting that CCR6^+^ILC3 may play a role in RA pathogenesis through the production of Th17 cytokines such as IL-17 and IL-22 (36). Finally, Yang et al. described that RA patients with stable disease depicted decreased ILC1 and increased ILC2 proportion in the peripheral blood compared with healthy controls and with patients with active disease, while both active and stable RA patients had a decreased percentage of ILC3 (33). A positive correlation between disease activity and ILC1 proportion was also found, while there was a negative correlation between ILC2 percentage and disease activity (33). This suggests that ILC2 may counterbalance the proinflammatory effect of ILC1 through the production of IL-13 (75, 76), which has been shown to have anti-inflammatory effect on synovitis in rheumatoid arthritis (77).

## Discussion

Since their initial description 10 years ago, ILC have been increasingly recognized as important players in the immune response, but their role in human autoimmune diseases remains controversial. Currently available data suggest that they could be useful as biomarkers of disease severity or response to treatment.

A major limitation to identify the role of ILC in human diseases is related to the fact that ILC are tissue-resident cells. Access to barrier site requests invasive biopsies, which are not easy to be routinely performed. Therefore, most studies are limited to the examination of cells from the peripheral blood, where ILC are only present at low abundance and where they display a phenotype that might differ from their tissue-resident counterpart. Technically, examination of restricted subpopulations of ILC may be difficult due to the important number of markers needed to identify these populations. Recent advances in single cell mass cytometry and flow cytometry that allow the examination of high amount of parameters in limited biological samples should facilitate future studies on the subject.

Therapeutic approaches targeting ILC are challenging, because ILC are highly heterogeneous. Moreover, no specific markers for ILC have been identified to date, making it difficult to develop a drug that specifically targets ILC or ILC subsets. Accordingly, the border between pathogenic versus beneficial role of ILC is not always obvious. ILC2, for example, seem to be pathogenic in atopic dermatitis, but in a mouse model of RA, they foster the resolution of inflammation (1, 78). Since ILC exhibit an important plasticity that depends on their microenvironment, targeting cytokines or soluble factors involved in their differentiation and maintenance would likely affect ILC subpopulation distribution and alter diseases course. Recent evidence, for example, showed that patients with inflammatory bowel disease exhibit an altered distribution of ILC subsets in the gut and blood during active disease. This anomaly is partly restored after treatment with the anti-IL12/23 monoclonal antibody ustekinumab (79). Overall, future studies are warranted to explore the role of ILC in human diseases, because currently available data remain largely descriptive and functional data are lacking.

## Author Contributions

AC, MH, NF, MK, and DC researched the data for the article. AC, MK, and DC wrote the manuscript. AC, MH, NF, MK, and DC reviewed the manuscript. All authors accepted the finale version of the manuscript

## Funding

This study received funding from the Swiss National Science Foundation (Ambizione PZ00P3_173950 to DC) and a grant from the Novartis Foundation for Medical-Biological Research (to DC). The funder was not involved in the study design, collection, analysis, interpretation of data, the writing of this article, or the decision to submit it for publication.

## Conflict of Interest

The authors declare that the research was conducted in the absence of any commercial or financial relationships that could be construed as a potential conflict of interest.

## Publisher’s Note

All claims expressed in this article are solely those of the authors and do not necessarily represent those of their affiliated organizations, or those of the publisher, the editors and the reviewers. Any product that may be evaluated in this article, or claim that may be made by its manufacturer, is not guaranteed or endorsed by the publisher.

## References

[1] Ebbo M Crinier A Vely F Vivier E. Innate Lymphoid Cells: Major Players in Inflammatory Diseases. Nat Rev Immunol (2017) 17(11):665–78. doi: 10.1038/nri.2017.86 28804130

[2] Spits H Artis D Colonna M Diefenbach A Di Santo JP Eberl G. Innate Lymphoid Cells–A Proposal for Uniform Nomenclature. Nat Rev Immunol (2013) 13(2):145–9. doi: 10.1038/nri3365 23348417

[3] Vivier E Artis D Colonna M Diefenbach A Di Santo JP Eberl G. Innate Lymphoid Cells: 10 Years on. Cell (2018) 174(5):1054–66. doi: 10.1016/j.cell.2018.07.017 30142344

[4] Cella M Gamini R Secca C Collins PL Zhao S Peng V. Subsets of ILC3-ILC1-Like Cells Generate a Diversity Spectrum of Innate Lymphoid Cells in Human Mucosal Tissues. Nat Immunol (2019) 20(8):980–91. doi: 10.1038/s41590-019-0425-y PMC6685551 31209406

[5] Xu W Cherrier DE Chea S Vosshenrich C Serafini N Petit M. An Id2(RFP)-Reporter Mouse Redefines Innate Lymphoid Cell Precursor Potentials. Immunity (2019) 50(4):1054–68.e3. doi: 10.1016/j.immuni.2019.02.022 30926235 PMC6477155

[6] Klose CSN Flach M Mohle L Rogell L Hoyler T Ebert K. Differentiation of Type 1 ILCs From a Common Progenitor to All Helper-Like Innate Lymphoid Cell Lineages. Cell (2014) 157(2):340–56. doi: 10.1016/j.cell.2014.03.030 24725403

[7] Scoville SD Mundy-Bosse BL Zhang MH Chen L Zhang X Keller KA. A Progenitor Cell Expressing Transcription Factor RORgammat Generates All Human Innate Lymphoid Cell Subsets. Immunity (2016) 44(5):1140–50. doi: 10.1016/j.immuni.2016.04.007 PMC4893782 27178467

[8] Carson WE Giri JG Lindemann MJ Linett ML Ahdieh M Paxton R. Interleukin (IL) 15 Is a Novel Cytokine That Activates Human Natural Killer Cells *via* components of the IL-2 receptor. J Exp Med (1994) 180(4):1395–403. doi: 10.1084/jem.180.4.1395 PMC2191697 7523571

[9] Fuchs A Vermi W Lee JS Lonardi S Gilfillan S Newberry RD. Intraepithelial Type 1 Innate Lymphoid Cells Are a Unique Subset of IL-12- and IL-15-Responsive IFN-Gamma-Producing Cells. Immunity (2013) 38(4):769–81. doi: 10.1016/j.immuni.2013.02.010 PMC3634355 23453631

[10] Kabata H Moro K Fukunaga K Suzuki Y Miyata J Masaki K. Thymic Stromal Lymphopoietin Induces Corticosteroid Resistance in Natural Helper Cells During Airway Inflammation. Nat Commun (2013) 4:2675. doi: 10.1038/ncomms3675 24157859

[11] Mjosberg J Bernink J Golebski K Karrich JJ Peters CP Blom B. The Transcription Factor GATA3 Is Essential for the Function of Human Type 2 Innate Lymphoid Cells. Immunity (2012) 37(4):649–59. doi: 10.1016/j.immuni.2012.08.015 23063330

[12] Constantinides MG McDonald BD Verhoef PA Bendelac A. A Committed Precursor to Innate Lymphoid Cells. Nature (2014) 508(7496):397–401. doi: 10.1038/nature13047 24509713 PMC4003507

[13] Freud AG Yokohama A Becknell B Lee MT Mao HC Ferketich AK. Evidence for Discrete Stages of Human Natural Killer Cell Differentiation *In Vivo* . J Exp Med (2006) 203(4):1033–43. doi: 10.1084/jem.20052507 PMC2118285 16606675

[14] Seillet C Mielke LA Amann-Zalcenstein DB Su S Gao J Almeida FF. Deciphering the Innate Lymphoid Cell Transcriptional Program. Cell Rep (2016) 17(2):436–47. doi: 10.1016/j.celrep.2016.09.025 27705792

[15] Yokota Y Mansouri A Mori S Sugawara S Adachi S Nishikawa S. Development of Peripheral Lymphoid Organs and Natural Killer Cells Depends on the Helix-Loop-Helix Inhibitor Id2. Nature (1999) 397(6721):702–6. doi: 10.1038/17812 10067894

[16] Gordon SM Chaix J Rupp LJ Wu J Madera S Sun JC. The Transcription Factors T-Bet and Eomes Control Key Checkpoints of Natural Killer Cell Maturation. Immunity (2012) 36(1):55–67. doi: 10.1016/j.immuni.2011.11.016 22261438 PMC3381976

[17] Renoux VM Zriwil A Peitzsch C Michaelsson J Friberg D Soneji S. Identification of a Human Natural Killer Cell Lineage-Restricted Progenitor in Fetal and Adult Tissues. Immunity (2015) 43(2):394–407. doi: 10.1016/j.immuni.2015.07.011 26287684

[18] Flommersfeld S Bottcher JP Ersching J Flossdorf M Meiser P Pachmayr LO. Fate Mapping of Single NK Cells Identifies a Type 1 Innate Lymphoid-Like Lineage That Bridges Innate and Adaptive Recognition of Viral Infection. Immunity (2021) 54(10):2288–304.e7. doi: 10.1016/j.immuni.2021.08.002 34437840 PMC8528403

[19] Guia S Narni-Mancinelli E. Helper-Like Innate Lymphoid Cells in Humans and Mice. Trends Immunol (2020) 41(5):436–52. doi: 10.1016/j.it.2020.03.002 32223931

[20] Meininger I Carrasco A Rao A Soini T Kokkinou E Mjosberg J. Tissue-Specific Features of Innate Lymphoid Cells. Trends Immunol (2020) 41(10):902–17. doi: 10.1016/j.it.2020.08.009 32917510

[21] Riggan L Freud AG O’Sullivan TE. True Detective: Unraveling Group 1 Innate Lymphocyte Heterogeneity. Trends Immunol (2019) 40(10):909–21. doi: 10.1016/j.it.2019.08.005 PMC6823149 31500958

[22] Di Censo C Marotel M Mattiola I Müller L Scarno G Pietropaolo G. Granzyme A and CD160 Expression Delineates ILC1 With Graded Functions in the Mouse Liver. Eur J Immunol (2021) 51(11):2568–75. doi: 10.1002/eji.202149209 PMC9292164 34347289

[23] Bal SM Golebski K Spits H. Plasticity of Innate Lymphoid Cell Subsets. Nat Rev Immunol (2020) 20(9):552–65. doi: 10.1038/s41577-020-0282-9 32107466

[24] Tominaga S. A Putative Protein of a Growth Specific cDNA From BALB/c-3T3 Cells Is Highly Similar to the Extracellular Portion of Mouse Interleukin 1 Receptor. FEBS Lett (1989) 258(2):301–4. doi: 10.1016/0014-5793(89)81679-5 2532153

[25] Roan F Stoklasek TA Whalen E Molitor JA Bluestone JA Buckner JH. CD4+ Group 1 Innate Lymphoid Cells (ILC) Form a Functionally Distinct ILC Subset That Is Increased in Systemic Sclerosis. J Immunol (2016) 196(5):2051–62. doi: 10.4049/jimmunol.1501491 PMC4761490 26826243

[26] Roan F Stoklasek TA Whalen E Molitor JA Bluestone JA Buckner JH. Correction: CD4+ Group 1 Innate Lymphoid Cells (ILC) Form a Functionally Distinct ILC Subset That Is Increased in Systemic Sclerosis. J Immunol (2016) 196(9):3966. doi: 10.4049/jimmunol.1600364 27183651

[27] Blokland SLM van den Hoogen LL Leijten EFA Hartgring SAY Fritsch R Kruize AA. Increased Expression of Fas on Group 2 and 3 Innate Lymphoid Cells Is Associated With an Interferon Signature in Systemic Lupus Erythematosus and Sjogren’s Syndrome. Rheumatol (Oxford) (2019) 58(10):1740–45. doi: 10.1093/rheumatology/kez116 31220315

[28] Guo C Zhou M Zhao S Zhao S Huang Y Wang S. Innate Lymphoid Cell Disturbance With Increase in ILC1 in Systemic Lupus Erythematosus. Clin Immunol (2019) 202:49–58. doi: 10.1016/j.clim.2019.03.008 30926441 PMC8191378

[29] Jiang Y Zhao Y Liu Y Huang Q Meng W Xu H. Imbalanced Innate Lymphoid Cells Are Associated With Disease Activity and Arthritis Involvement in Patients With Systemic Lupus Erythematosus. Arch Rheumatol (2020) 35(4):521–32. doi: 10.46497/ArchRheumatol.2020.7440 PMC7945710 33758809

[30] Hou M Liu S. Innate Lymphoid Cells Are Increased in Systemic Lupus Erythematosus. Clin Exp Rheumatol (2019) 37(4):676–79. 30789153

[31] Braudeau C Amouriaux K Neel A Herbreteau G Salabert N Rimbert M. Persistent Deficiency of Circulating Mucosal-Associated Invariant T (MAIT) Cells in ANCA-Associated Vasculitis. J Autoimmun (2016) 70:73–9. doi: 10.1016/j.jaut.2016.03.015 27102145

[32] Rodriguez-Carrio J Hahnlein JS Ramwadhdoebe TH Semmelink JF Choi IY van Lienden KP. Brief Report: Altered Innate Lymphoid Cell Subsets in Human Lymph Node Biopsy Specimens Obtained During the At-Risk and Earliest Phases of Rheumatoid Arthritis. Arthritis Rheumatol (2017) 69(1):70–6. doi: 10.1002/art.39811 PMC6681066 27428460

[33] Yang F Luo X Zhu W Li J Zheng Z Zhu P. Dysregulation of Innate Lymphoid Cells in Patients With Active Rheumatoid Arthritis and Mice With Collagen-Induced Arthritis. Mediators Inflamm (2021) 2021:1915068. doi: 10.1155/2021/1915068 33688303 PMC7920742

[34] Wohlfahrt T Usherenko S Englbrecht M Dees C Weber S Beyer C. Type 2 Innate Lymphoid Cell Counts Are Increased in Patients With Systemic Sclerosis and Correlate With the Extent of Fibrosis. Ann Rheum Dis (2016) 75(3):623–6. doi: 10.1136/annrheumdis-2015-207388 26338035

[35] Laurent P Allard B Manicki P Jolivel V Levionnois E Jeljeli M. TGFbeta Promotes Low IL10-Producing ILC2 With Profibrotic Ability Involved in Skin Fibrosis in Systemic Sclerosis. Ann Rheum Dis (2021) 80(12):1594–603. doi: 10.1136/annrheumdis-2020-219748 PMC8600612 34285051

[36] Takaki-Kuwahara A Arinobu Y Miyawaki K Yamada H Tsuzuki H Irino K. CCR6+ Group 3 Innate Lymphoid Cells Accumulate in Inflamed Joints in Rheumatoid Arthritis and Produce Th17 Cytokines. Arthritis Res Ther (2019) 21(1):198. doi: 10.1186/s13075-019-1984-x 31470891 PMC6716915

[37] Galy A Travis M Cen D Chen B. Human T, B, Natural Killer, and Dendritic Cells Arise From a Common Bone Marrow Progenitor Cell Subset. Immunity (1995) 3(4):459–73. doi: 10.1016/1074-7613(95)90175-2 7584137

[38] Scoville SD Freud AG Caligiuri MA. Cellular Pathways in the Development of Human and Murine Innate Lymphoid Cells. Curr Opin Immunol (2019) 56:100–06. doi: 10.1016/j.coi.2018.11.003 PMC7285385 30579240

[39] Abel AM Yang C Thakar MS Malarkannan S. Natural Killer Cells: Development, Maturation, and Clinical Utilization. Front Immunol (2018) 9:1869. doi: 10.3389/fimmu.2018.01869 30150991 PMC6099181

[40] Dogra P Rancan C Ma W Toth M Senda T Carpenter DJ. Tissue Determinants of Human NK Cell Development, Function, and Residence. Cell (2020) 180(4):749–63.e13. doi: 10.1016/j.cell.2020.01.022 32059780 PMC7194029

[41] Mjosberg JM Trifari S Crellin NK Peters CP van Drunen CM Piet B. Human IL-25- and IL-33-Responsive Type 2 Innate Lymphoid Cells Are Defined by Expression of CRTH2 and CD161. Nat Immunol (2011) 12(11):1055–62. doi: 10.1038/ni.2104 21909091

[42] Martinez-Gonzalez I Matha L Steer CA Ghaedi M Poon GFT Takei F. Allergen-Experienced Group 2 Innate Lymphoid Cells Acquire Memory-Like Properties and Enhance Allergic Lung Inflammation. Immunity (2016) 45(1):198–208. doi: 10.1016/j.immuni.2016.06.017 27421705

[43] Kramer B Goeser F Lutz P Glassner A Boesecke C Schwarze-Zander C. Compartment-Specific Distribution of Human Intestinal Innate Lymphoid Cells Is Altered in HIV Patients Under Effective Therapy. PLoS Pathog (2017) 13(5):e1006373. doi: 10.1371/journal.ppat.1006373 28505204 PMC5444854

[44] Magri G Cerutti A. Role of Group 3 Innate Lymphoid Cells in Antibody Production. Curr Opin Immunol (2015) 33:36–42. doi: 10.1016/j.coi.2015.01.008 25621842 PMC4488900

[45] Bernink JH Krabbendam L Germar K de Jong E Gronke K Kofoed-Nielsen M. Interleukin-12 and -23 Control Plasticity of CD127(+) Group 1 and Group 3 Innate Lymphoid Cells in the Intestinal Lamina Propria. Immunity (2015) 43(1):146–60. doi: 10.1016/j.immuni.2015.06.019 26187413

[46] Yudanin NA Schmitz F Flamar AL Thome JJC Tait Wojno E Moeller JB. Spatial and Temporal Mapping of Human Innate Lymphoid Cells Reveals Elements of Tissue Specificity. Immunity (2019) 50(2):505–19.e4. doi: 10.1016/j.immuni.2019.01.012 30770247 PMC6594374

[47] Bar-Ephraim YE Cornelissen F Papazian N Konijn T Hoogenboezem RM Sanders MA. Cross-Tissue Transcriptomic Analysis of Human Secondary Lymphoid Organ-Residing ILC3s Reveals a Quiescent State in the Absence of Inflammation. Cell Rep (2017) 21(3):823–33. doi: 10.1016/j.celrep.2017.09.070 29045847

[48] Scandella E Bolinger B Lattmann E Miller S Favre S Littman DR. Restoration of Lymphoid Organ Integrity Through the Interaction of Lymphoid Tissue-Inducer Cells With Stroma of the T Cell Zone. Nat Immunol (2008) 9(6):667–75. doi: 10.1038/ni.1605 18425132

[49] Kim MY Kim KS McConnell F Lane P. Lymphoid Tissue Inducer Cells: Architects of CD4 Immune Responses in Mice and Men. Clin Exp Immunol (2009) 157(1):20–6. doi: 10.1111/j.1365-2249.2009.03932.x PMC2710588 19659766

[50] Shikhagaie MM Bjorklund AK Mjosberg J Erjefält JS Cornelissen AS Ros XR. Neuropilin-1 Is Expressed on Lymphoid Tissue Residing LTi-Like Group 3 Innate Lymphoid Cells and Associated With Ectopic Lymphoid Aggregates. Cell Rep (2017) 18(7):1761–73. doi: 10.1016/j.celrep.2017.01.063 PMC5318658 28199847

[51] Wang S Xia P Chen Y Qu Y Xiong Z Ye B. Regulatory Innate Lymphoid Cells Control Innate Intestinal Inflammation. Cell (2017) 171(1):201–16.e18. doi: 10.1016/j.cell.2017.07.027 28844693

[52] Bando JK Gilfillan S Di Luccia B Fachi JL Secca C Cella M. ILC2s Are the Predominant Source of Intestinal ILC-Derived IL-10. J Exp Med (2020) 217(2):1–9. doi: 10.1084/jem.20191520 PMC7041711 31699824

[53] Cao Q Wang R Wang Y Niu Z Chen T Wang C. Regulatory Innate Lymphoid Cells Suppress Innate Immunity and Reduce Renal Ischemia/Reperfusion Injury. Kidney Int (2020) 97(1):130–42. doi: 10.1016/j.kint.2019.07.019 31685310

[54] O’Connor MH Muir R Chakhtoura M Fang M Moysi E Moir S. A Follicular Regulatory Innate Lymphoid Cell Population Impairs Interactions Between Germinal Center Tfh and B Cells. Commun Biol (2021) 4(1):563. doi: 10.1038/s42003-021-02079-0 33980982 PMC8115650

[55] Barsotti S Orlandi M Codullo V Di Battista M Lepri G Della Rossa A. One Year in Review 2019: Systemic Sclerosis. Clin Exp Rheumatol (2019) 37 Suppl 119(4):3–14. 31587697

[56] Perelas A Arrossi AV Highland KB. Pulmonary Manifestations of Systemic Sclerosis and Mixed Connective Tissue Disease. Clin Chest Med (2019) 40(3):501–18. doi: 10.1016/j.ccm.2019.05.001 31376887

[57] Denton CP Khanna D. Systemic Sclerosis. Lancet (2017) 390(10103):1685–99. doi: 10.1016/S0140-6736(17)30933-9 28413064

[58] Asano Y. The Pathogenesis of Systemic Sclerosis: An Understanding Based on a Common Pathologic Cascade Across Multiple Organs and Additional Organ-Specific Pathologies. J Clin Med (2020) 9(9):1–27. doi: 10.3390/jcm9092687 PMC7565034 32825112

[59] De Martinis M Ciccarelli F Sirufo MM Ginaldi L. An Overview of Environmental Risk Factors in Systemic Sclerosis. Expert Rev Clin Immunol (2016) 12(4):465–78. doi: 10.1586/1744666X.2016.1125782 26610037

[60] Kim D Peck A Santer D Patole P Schwartz SM Molitor JA. Induction of Interferon-Alpha by Scleroderma Sera Containing Autoantibodies to Topoisomerase I: Association of Higher Interferon-Alpha Activity With Lung Fibrosis. Arthritis Rheum (2008) 58(7):2163–73. doi: 10.1002/art.23486 18576347

[61] Wen FQ Kohyama T Liu X Zhu YK Wang H Kim HJ. Interleukin-4- and Interleukin-13-Enhanced Transforming Growth Factor-Beta2 Production in Cultured Human Bronchial Epithelial Cells Is Attenuated by Interferon-Gamma. Am J Respir Cell Mol Biol (2002) 26(4):484–90. doi: 10.1165/ajrcmb.26.4.4784 11919085

[62] Lee HS Kooshesh F Sauder DN Kondo S. Modulation of TGF-Beta 1 Production From Human Keratinocytes by UVB. Exp Dermatol (1997) 6(2):105–10. doi: 10.1111/j.1600-0625.1997.tb00155.x 9209893

[63] Wang L Tang J Yang X Zanvit P Cui K Ku WL. TGF-Beta Induces ST2 and Programs ILC2 Development. Nat Commun (2020) 11(1):35. doi: 10.1038/s41467-019-13734-w 31911623 PMC6946674

[64] Tsokos GC Lo MS Costa Reis P Sullivan KE. New Insights Into the Immunopathogenesis of Systemic Lupus Erythematosus. Nat Rev Rheumatol (2016) 12(12):716–30. doi: 10.1038/nrrheum.2016.186 27872476

[65] Bernink JH Mjosberg J Spits H. Human ILC1: To Be or Not to be. Immunity (2017) 46(5):756–57. doi: 10.1016/j.immuni.2017.05.001 PMC5441993 28514676

[66] Jennette JC. Overview of the 2012 Revised International Chapel Hill Consensus Conference Nomenclature of Vasculitides. Clin Exp Nephrol (2013) 17(5):603–06. doi: 10.1007/s10157-013-0869-6 PMC4029362 24072416

[67] Kronbichler A Lee KH Denicolo S Choi D Lee H Ahn D. Immunopathogenesis of ANCA-Associated Vasculitis. Int J Mol Sci (2020) 21(19):1–27. doi: 10.3390/ijms21197319 PMC7584042 33023023

[68] Kallenberg CG. Advances in Pathogenesis and Treatment of ANCA-Associated Vasculitis. Discovery Med (2014) 18(99):195–201. 25336033

[69] McInnes IB Schett G. The Pathogenesis of Rheumatoid Arthritis. N Engl J Med (2011) 365(23):2205–19. doi: 10.1056/NEJMra1004965 22150039

[70] Rantapaa-Dahlqvist S de Jong BA Berglin E Hallmans G Wadell G Stenlund H. Antibodies Against Cyclic Citrullinated Peptide and IgA Rheumatoid Factor Predict the Development of Rheumatoid Arthritis. Arthritis Rheum (2003) 48(10):2741–9. doi: 10.1002/art.11223 14558078

[71] Guo Q Wang Y Xu D Nossent J Pavlos NJ Xu J. Rheumatoid Arthritis: Pathological Mechanisms and Modern Pharmacologic Therapies. Bone Res (2018) 6:15. doi: 10.1038/s41413-018-0016-9 29736302 PMC5920070

[72] Symmons DP Bankhead CR Harrison BJ Brennan P Silman AJ Barrett EM. Blood Transfusion, Smoking, and Obesity as Risk Factors for the Development of Rheumatoid Arthritis: Results From a Primary Care-Based Incident Case-Control Study in Norfolk, England. Arthritis Rheum (1997) 40(11):1955–61. doi: 10.1002/art.1780401106 9365083

[73] Dalbeth N. Callan MF. A Subset of Natural Killer Cells Is Greatly Expanded Within Inflamed Joints. Arthritis Rheum (2002) 46(7):1763–72. doi: 10.1002/art.10410 12124859

[74] Fang W Zhang Y Chen Z. Innate Lymphoid Cells in Inflammatory Arthritis. Arthritis Res Ther (2020) 22(1):25. doi: 10.1186/s13075-020-2115-4 32051038 PMC7017550

[75] Mindt BC Fritz JH Duerr CU. Group 2 Innate Lymphoid Cells in Pulmonary Immunity and Tissue Homeostasis. Front Immunol (2018) 9:840. doi: 10.3389/fimmu.2018.00840 29760695 PMC5937028

[76] Sugita K Steer CA Martinez-Gonzalez I Altunbulakli C Morita H Castro-Giner F. Type 2 Innate Lymphoid Cells Disrupt Bronchial Epithelial Barrier Integrity by Targeting Tight Junctions Through IL-13 in Asthmatic Patients. J Allergy Clin Immunol (2018) 141(1):300–10 e11. doi: 10.1016/j.jaci.2017.02.038 28392332

[77] Isomaki P Luukkainen R Toivanen P Punnonen J. The Presence of Interleukin-13 in Rheumatoid Synovium and Its Antiinflammatory Effects on Synovial Fluid Macrophages From Patients With Rheumatoid Arthritis. Arthritis Rheum (1996) 39(10):1693–702. doi: 10.1002/art.1780391012 8843860

[78] Rauber S Luber M Weber S Maul L Soare A Wohlfahrt T. Resolution of Inflammation by Interleukin-9-Producing Type 2 Innate Lymphoid Cells. Nat Med (2017) 23(8):938–44. doi: 10.1038/nm.4373 PMC5575995 28714991

[79] Creyns B Jacobs I Verstockt B Cremer J Ballet V Vandecasteele R. Biological Therapy in Inflammatory Bowel Disease Patients Partly Restores Intestinal Innate Lymphoid Cell Subtype Equilibrium. Front Immunol (2020) 11:1847. doi: 10.3389/fimmu.2020.01847 32983101 PMC7481382

